# Investigation of Multi-Plane Scheme for Compensation of Fringe Effect of Electrical Resistance Tomography Sensor †

**DOI:** 10.3390/s19143132

**Published:** 2019-07-16

**Authors:** Wenbin Tian, Xiaofeng Liang, Xiaolei Qu, Jiangtao Sun, Shuo Gao, Lijun Xu, Wuqiang Yang

**Affiliations:** 1 School of Instrumentation and Optoelectronic Engineering, Beihang University, Beijing 100191, China; 2 Beijing Advanced Innovation Center for Big Data-Based Precision Medicine, Beihang University, Beijing 100191, China; 3 School of Electrical and Electronic Engineering, The University of Manchester, Manchester M13 9PL, UK

**Keywords:** electrical resistance tomography, fringe effect, multiple-plane ERT sensor

## Abstract

Conventional electrical resistance tomography (ERT) sensors suffer from the fringe effect, i.e., severe distortion of the electric field on both ends of the measurement electrodes, leading to a 3D sensing region for a 2D sensor. As a result, the objects outside an ERT sensor plane affect the sensing and hence image, i.e., deteriorating the image quality. To address this issue, a multiple-plane ERT sensor scheme is proposed in this paper. With this scheme, auxiliary sensor planes are used to provide references for the fringe effect of the measurement plane, for compensation by subtracting the weighed influence of the fringe effect. Simulation results show that the proposed scheme, either three-plane or two-plane sensor, can compensate for the fringe effect induced by objects outside the measurement plane with a variety of axial object distributions, i.e., several non-conductive bars or conductive bars placed at different cross-sectional and axial positions inside the sensor. Experiments were carried out. Images obtained with single-plane and multiple-plane ERT sensors are compared, and the proposed compensation scheme has been hence verified.

## 1. Introduction

Electrical resistance tomography (ERT) is an imaging technique used to visualize and measure the distribution of object(s) or material with different electrical properties within an imaging plane or volume of interest using a multi-electrode sensor. In the conventional ERT, the current-injection and voltage-measurement strategy is adopted. To interrogate an imaging area of interest, an AC current is applied to one pair of electrodes sequentially while other electrodes are floating, and the inter-electrode resistance is measured. This process continues until a complete set of data is taken. Finally, an image of object or material distribution is reconstructed according to the measurement data using an appropriate algorithm.

ERT has found many applications, from biomedical imaging to multiphase flow measurement in industrial processes, featured by providing 2D or even 3D images. Recently, research groups in the NC State University and University of Eastern Finland have obtained promising results for new applications of ERT, such as concrete damage detection and measurement of unsaturated moisture flow in cementitious materials [1,2,3,4]. For imaging and monitoring fast changing multiphase flows, such as in bubble columns [5,6,7], 2D imaging is preferred as it is much simpler and faster than 3D imaging because with 3D imaging the measurement data are incomplete, the inverse problem is more severely ill-posed and ill-conditioned and the computation time is much longer, than 2D imaging [8]. However, the distribution of electric field of an ERT sensor is inherently 3D and it is necessary to represent the 3D distribution by an approximated 2D one. Due to the “soft-field” nature of ERT, the 3D electric field depends on the object distribution being measured. Two conditions should be satisfied to allow the 3D distribution to be simplified into 2D with tolerable errors: (1) The electric field should be confined within an ERT sensor and axially uniform, which is subject to the sensor structure, especially the length of the electrodes [9]; and (2) the object distribution is axially uniform. In conventional ERT, however, “pin” electrodes are used, which cannot generate an axially uniform electric field. This situation would become worse, i.e., severe distortion of electric field at both ends of the electrodes or so-called fringe effect would occur if the object distribution is axially non-uniform, especially when there exist considerably large objects in the region close to the electrode edge.

According to the literature, the fringe effect of ERT sensors and its influences on 2D imaging have been investigated [8,9,10,11,12,13]. However, only a few of them discussed about how to reduce the fringe effect to improve the image quality [8,9]. Sun and Yang evaluated the fringe effect of an ERT sensor for typical distributions with objects of different diameters, lengths, and conductivities at different cross-sectional and axial positions by simulation and experiment [8]. It was concluded that if an object is further away from the electrode plane or it is shorter, the less severe the fringe effect is. When an object of a certain length moves away from the electrode plane, the position of the reconstructed object approaches to the center of the sensor, due to the larger distortion of fringe fields in the distant location. A simple direct scaling method was proposed to compensate for the fringe effect, which was proved to be effective in certain cases [8]. However, in some complicated and extreme circumstances, where the fringe effect distorts the signal too much, it would be difficult to implement the direct scaling method to compensate for the fringe effect. 

This paper presents a new approach to compensating for the fringe effect of ERT sensors. One or two auxiliary electrode planes adjacent to the measurement electrode plane is/are used to sense the fringe effect induced by objects outside the measurement plane. Therefore, this is a multi-plane ERT sensor scheme. Compensation is achieved by weighed subtraction of the sensed fringe effect from the measurement data from the measurement plan. The feasibility and effectiveness of the proposed method is validated by simulation and experiment by several scenarios with varied object settings outside the measurement plane, such as the number and geometry of objects and distance away from the measurement plane. The influence of the proposed method on imaging of objects inside the measurement plane are demonstrated, showing that over-estimation of the size of objects can be alleviated by linear image reconstruction methods, in particular the Landweber iteration. 

## 2. Image Reconstruction 

A variety of reconstruction algorithms have been reported in the literature [14]. Among them, linear back-projection (LBP) and the Landweber iteration are the most popular. The Landweber iteration is an iterative method and can produce quantitative images. It is used in this paper for image reconstruction. To implement the Landweber iteration, sensitivity maps of an ERT sensor and normalization of measured data are needed. A 2D sensitivity map is composed by the sensitivity of electrode pairs i and j to the conductivity change of the pixel at position (x,y) with an area of P(x,y) [15]:

(1) $$S_{i,j}\left(x,y\right)=\int_{P\left(x,y\right)}\frac{E_{i}\left(x,y\right)}{I_{i}}\cdot \frac{E_{j}\left(x,y\right)}{I_{j}}dxdy,$$

where $E_{i}\left(x,y\right)$ and $E_{j}\left(x,y\right)$ are the electric field strength at (x,y) when the *i*th and *j*th electrode pairs are injected with currents $I_{i}$ and $I_{j}$*,* respectively for excitation in turn.

For ERT, the measured voltage differences are normalized by calculating their relative changes with respect to the reference voltage differences, which are obtained when an ERT sensor is filled with a conductive background medium. It can be expressed as [15,16]:

(2) $$\lambda \left(i,j\right)=\frac{V_{m}\left(i,j\right)-V_{r}\left(i,j\right)}{V_{r}\left(i,j\right)},$$

where λ(i,j) is the normalized change in voltage difference for injection electrode pair i and measurement electrode pair j, and $V_{m}\left(i,j\right)$ and $V_{r}\left(i,j\right)$ are the measured and reference voltage difference for injection electrode pair i and measurement electrode pair j*,* respectively.

Landweber iteration method [17] is derived from the steepest gradient descent method in the optimization theory. To improve the convergence rate, the original Landweber iteration is modified to be [14]:

(3) $$g_{k+1}=P\left[g_{k}-\alpha_{k}S^{T}\left(Sg_{k}-\lambda \right)\right],$$

where $g_{k}$ and $g_{k+1}$ are the normalized permittivity vector for *k*th and (*k*+ 1)th iteration, $\alpha_{k}$ is the gain or relaxation factor, which is used to determine the convergence rate. P is a projection operator:

(4) $$P\left[f\left(x\right)\right]=\{\begin{array}{cc} 0 & if\;f\left(x\right)<0\\ f\left(x\right) & if\;0<f\left(x\right)<1\\ 1 & if\;f\left(x\right)>1 \end{array}.$$

In the iteration process, the relaxation factor $\alpha_{k}$ can be updated in each iteration as discussed by Liu et al. [18]. To start the Landweber iteration process, the initial permittivity distribution $g_{0}$ is normally reconstructed by LBP. Two criteria are employed to evaluate the performance of reconstruction algorithms, which are relative image error and correlation coefficient between the true image and the reconstructed image. Their definitions were given in [12]. 

## 3. Fringe Field Compensation Scheme

### 3.1. Analysis of Fringe Field Distribution

Many researchers considered the fringe effect between parallel electrode plates. Metodiey et al. analyzed the fringe fields between two finite parallel flat plates [19]. For two flat plates charged at +V_0_ and –V_0_, the electric field between them can be expressed by an implicit mapping [19]. The generated radial electric field *Ey* and longitudinal electric field *Ex* are defined as:

(5) $$E_{x}=\frac{2V_{0}}{d}\frac{e^{u}\sin v}{\left(1+2e^{u}\cos v+e^{2u}\right)},$$

(6) $$E_{y}=-\frac{2V_{0}}{d}\frac{e^{u}\cos v}{\left(1+2e^{u}\cos v+e^{2u}\right)},$$

where d is the distance between the plates. The *x* direction stands for the longitudinal direction along the plates, and the *y* direction is the direction perpendicular to the plates. Therefore, the radial and longitudinal electric fields can be obtained in terms of u and v based on the implicit mapping. 

**Figure 1:**
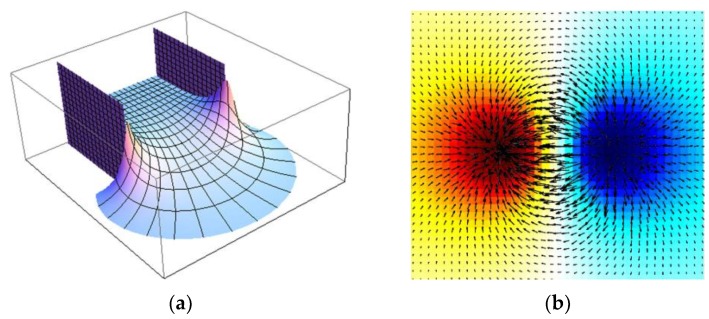
Static electric field or electrical current density distribution between two capacitive or resistive electrodes. (**a**) Magnitude of static electric field between parallel electrode plates [19]; (**b**) current density distribution between a pair of adjacent electrodes.

An analytical solution to the static electric field between two parallel plates was derived and shown in Figure 1a [19]. It is observed that the fringe electric fields between the parallel plates are distorted and decay in magnitude with the distance from the edge of the plates. According to a similar governing law, this kind of fringe effect also exists between resistive electrodes. Figure 1b shows the current density distribution between a pair of adjacent electrodes in an ERT sensor, obtained by finite element simulation. The further away from the electrode edge, the more decayed and distorted the fringe electric fields would be.

According to Equation (1), the sensitivity distribution is subject to the electric field distribution inside the sensing domain of an ERT sensor. Thus, the fringe effect at different axial positions can be observed in the sensitivity distributions in the corresponding plane above or below the measurement plane. The sensitivity maps of a conventional single-plane ERT sensor are generated to show the fringe effect at different axial positions between the adjacent or opposite electrode pair. In this case, the planes above the top end of electrodes by 0.5, 3, and 5 cm are selected for comparison. 

**Figure 2:**
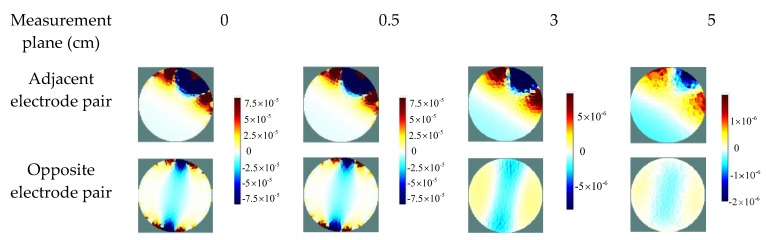
Sensitivity maps of a single-plane electrical resistance tomography (ERT) sensor at different axial positions between adjacent or opposite electrode pair.

Figure 2 shows the sensitivity distributions between the adjacent or opposite electrode pairs. Compared to the sensitivity distribution in the measurement plane, it is obvious that the sensitivity decreases when the plane is far away from the measurement plane. However, the sensitivity distribution between each electrode pair does not vary obviously when the selected plane is just above the measurement plane by 0.5 cm. Although the fringe sensitivity is not as large as in the measurement plane, the objects outside the measurement plane still affect the images if they are sufficiently large.

To quantify the similarity between the sensitivity distributions shown in Figure 2, one of the evaluation criteria for reconstructed images can be adopted, which is the correlation coefficient between sensitivity distributions in the measurement electrode plane and other selected axial plane. It is defined as:

(7) $$CC_{sd}=\frac{\sum_{i=1}^{N}({\hat{S}}_{i}-\bar{\hat{S}})(S_{i}-\bar{S})}{\sqrt{\sum_{i=1}^{N}{\left({\hat{S}}_{i}-\bar{\hat{S}}\right)}^{2}\sum_{i=1}^{N}{\left(S_{i}-\bar{S}\right)}^{2}}},$$

where S is the sensitivity distribution in the measurement electrode plane between a specified electrode pair,$\;\hat{S}$ is the sensitivity distribution in the selected plane above or below the measurement plane, and $\bar{S}$ and $\bar{\hat{S}}$ are the mean values of S and$\;\hat{S}$*,* respectively.

**Figure 3:**
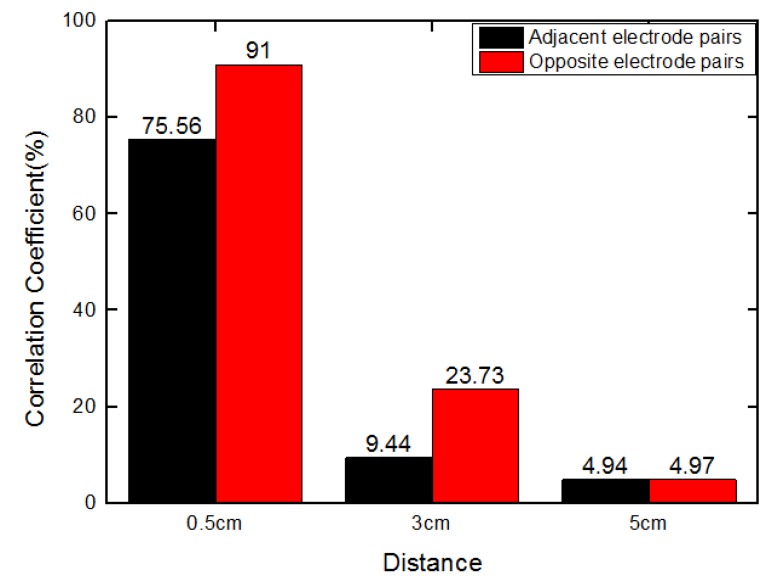
Correlation coefficient between sensitivity distributions in measurement electrode plane and other selected planes.

Figure 3 shows a decreased trend of the correlation coefficient, indicating that the similarity between sensitivity distributions becomes smaller when the selected plane is moving away from the measurement plane. However, it is undesirable for fringe effect compensation because the auxiliary electrode plane should have similar responses to the fringe effect (both in magnitude and pattern regarding the sensitivity distributions) as in the measurement plane to provide references for compensation. Therefore, the auxiliary electrode plane should be as close as possible to the measurement electrode plane. 

### 3.2. Compensation Scheme

Based on the above analysis, it is possible to attain the fringe effect induced by the objects outside the measurement plane. We propose the use of auxiliary electrode planes in an ERT sensor for compensation purpose, which is a multi-plane sensor scheme. With the multi-plane sensor, the objects outside the sensor can be sensed by each individual plane simultaneously. If electrode arrangements in the measurement plane and the auxiliary planes are same, it can be assumed that there is a proportional relationship between the sensed fringe effect by the measurement plane and an auxiliary plane when those electrode planes are sufficiently close to each other as discussed above, which can be expressed as:

(8) $$\lambda_{1}=k\cdot \lambda_{2}$$

where $\lambda_{1}$ and $\lambda_{2}$ are the sensed fringe effect by the measurement plane and an auxiliary plane, respectively. k is the proportional factor, which is application-dependent. Based on this assumption, the compensation scheme can be achieved by subtracting the weighed measurement data in the auxiliary plane from that in the measurement plane, which is described in detail in Section 4.1. To compensate for the fringe effect, three-plane and two-plane ERT sensor schemes are investigated in the following.

## 4. Simulation and Results

### 4.1. Three-Plane ERT Sensor Scheme

A three-plane ERT sensor scheme was proposed by Sun and Yang to compensate for fringe effect [8]. The three electrode planes of the ERT sensor can be denoted as the top, middle, and bottom planes. The middle plane is mainly for image reconstruction, while the other two planes are auxiliary planes for the compensation of fringe effect induced by objects outside the sensor plane. According to a 3D model for ERT [8,20], objects inside the ERT sensor can be sensed by all the three electrode planes if the same excitation signal and measurement strategy applied to all the three electrode planes simultaneously. Similarly, objects above or below the middle plane are sensed by the middle plane and top or bottom plane at the same time. As these three electrode planes are the same in geometry and structure, their responses to the same object inside their sensing ranges are correlated to each other. Then, the fringe effect induced by the objects above the middle plane may be compensated with the measurements in the top plane, while the fringe effect induced by objects below the middle plane may be compensated with the measurements in the top and bottom planes. The compensation can be made by subtracting the weighed measurement data before normalization in the top and bottom planes from the measurement data before normalization in the middle plane, as given by

(9) $$V_{ac}=V_{m}-WF*\left(V_{t}+V_{b}\right)$$

where $V_{ac}$ is the measured vector of potential differences after compensation, $V_{m}$, $V_{t}$, and $V_{b}$ are the measured vectors of potential differences in the middle, top, and bottom electrode planes, respectively, and WF is the weighting factor, which is a small positive scalar and initially determined based on trial-and-error. 

A three-plane ERT sensor is designed to verify the proposed method. The inner diameter of the sensor is 10 cm, and the number of the electrode in each plane is 16. The gap between adjacent planes is 5 mm. Unlike the driven guards in ERT sensors, measurements are also taken from the top and bottom planes in the same way as in the middle plane. With the adjacent measurement strategy, a pair of adjacent electrodes in the middle plane is injected a current signal, while the two pairs of adjacent electrodes in the top and bottom planes above and below this electrode pair are injected with almost the same current signal, respectively. Note that the electrodes in the same column are injected currents of the same polarity. Potential differences are measured in each electrode plane separately according to the measurement strategy. This process is repeated until all independent measurements in each electrode plane are taken. The number of independent measurements is determined by N(N − 3)/2, where N is the number of electrodes. In the simulation, each pair of differential currents has a peak-to-peak magnitude of 10 mA and exactly out of phase. The frequency of the injected AC current is 10 kHz. 

The measurement data after compensation are normalized using the reference data acquired in the middle plane with conductive background medium filling the sensor. Compensation is only effective in reducing the fringe effect induced by objects outside the ERT measurement sensor plane. For the axially non-uniform distribution with a single object inside the sensor, the fringe effect can be reduced by direct scaling [8]. Additionally, it was shown that the reconstruction algorithm a linear forward projection, e.g., Landweber iteration, tends to over-estimate the size of a non-conductive object being imaged when the distribution is axially uniform and this can be overcome with a forward operator based on a finite element method (FEM), which is computationally intensive [17]. For compensation purpose, it was found that the multi-plane sensor scheme is also effective to reduce this kind of over-estimation by linear forward projection (e.g., Landweber iteration) with less time consumption when the distribution is axially uniform. Therefore, this paper will examine the axially uniform distribution with a non-conductive object or axially non-uniform distribution with multiple non-conductive objects inside or outside the measurement sensor plane. Meanwhile, the same phantoms with conductive materials are also used in this case for comparison. Note that the conductivity of conductive materials is higher than that of background medium.

**Figure 4:**
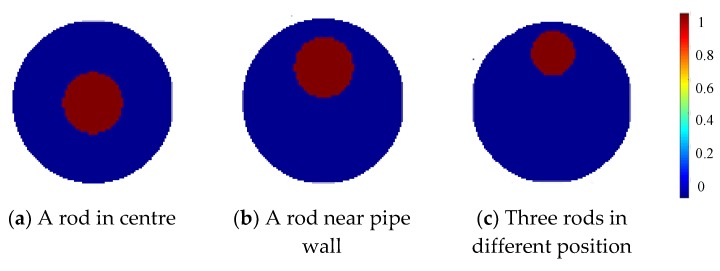
Normalized daxially uniform object distributions for simulation with three-plane ERT sensor.

Initial simulation was carried out to investigate the proposed three-plane sensor scheme. Three different setups were tested to evaluate the effectiveness of the three-plane ERT sensor in reducing the fringe effect and over-estimation by the Landweber iteration. The cross-sectional views of the normalized and true distributions for the three setups are shown in Figure 4.

**Figure 5:**
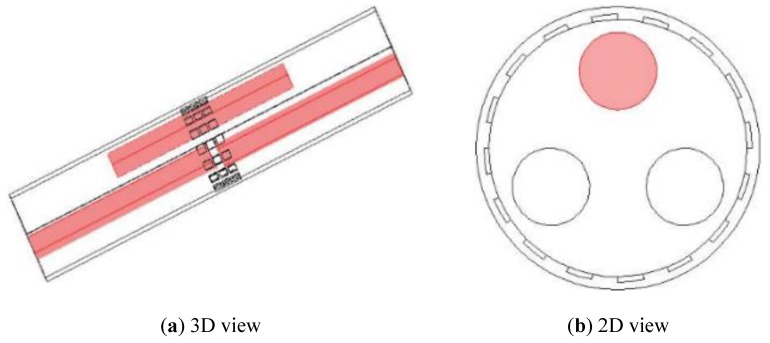
3D and 2D view of three rods in different axial and cross-sectional positions with only one inside middle plane for imaging.

In Figure 4c, three rods are distributed in different axial and cross-sectional positions, as shown in Figure 5a; but only the one marked with red color in Figure 5b is inside the middle plane for imaging with other two outside the middle plane. These three rods are of the same diameter and almost the same length. In all these setups, the rods are non-conductive with saline of conductivity 0.02 S/m as the background medium. The measured data in different planes are different but are correlated with each other. The fringe effect induced by objects outside the middle plane can be extracted from the measurements in the top and bottom planes. For the distribution in Figure 4a,b, the simulated potential differences acquired in the three sensor planes are similar to each other because the distribution is almost the same for all the three planes.

**Figure 6:**
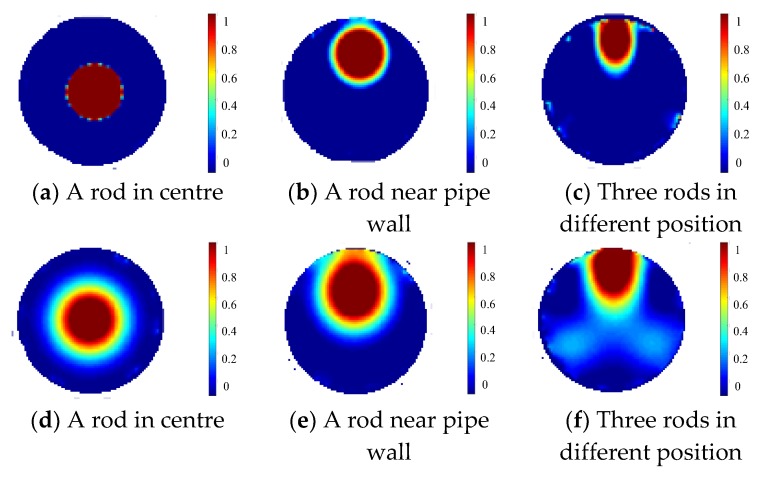
Reconstruction results for simulation setups with three-plane ERT sensor (**a**–**c**) or single-plane ERT sensor (**d**–**f**).

Using the normalized data after compensation, images are reconstructed for the above setups by the projected Landweber iteration as shown in Figure 6a–c. For comparison, the reconstruction results with a single-plane ERT sensor are shown in Figure 6d–f. Note that the single-plane ERT sensor has only the middle electrode plane in the three-plane ERT sensor with other geometry parameters unchanged. The weighting factors are 0.166, 0.101, 0.114 for the setups in Figure 6a–c, respectively, during the reconstruction. For comparison of quantity, the relative image errors and correlation coefficients of Figure 6a–f regarding the respective true distribution are listed in Table 1, as well as the settings of relaxation factor and the number of iterations for optimized reconstruction using the projected Landweber iteration. Note that the relaxation factor is updated in each iteration according to the linear search method proposed by Liu et al. [14]. Figure 6 and Table 1 also show that the over-estimation by the Landweber iteration and the fringe effect induced by objects outside the measurement plane can be substantially reduced with the three-plane ERT sensor scheme, especially in Figure 6c compared to Figure 6f. Note that the images of the two objects outside the middle sensor plane by the single-plane ERT sensor in Figure 6f are not very prominent with only three iterations. The grey level would be higher with more iterations, i.e., more severe artefacts in the reconstructed image. The iteration will stop when the image error in the current iteration becomes larger than the one in the previous iteration.

**Figure 7:**
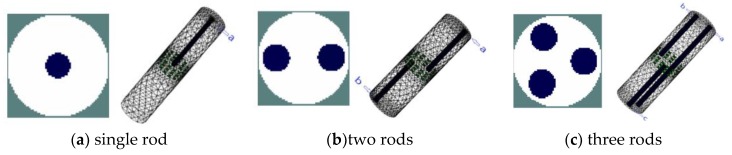
3D and 2D view of axially non-uniform rods in different axial and cross-sectional positions inside three-plane ERT sensor

To further investigate the effectiveness of the three-plane sensor scheme, more simulation was carried out for a variety of scenarios with varied object size, arrangement, and conductivity as well as sensor geometry. Note that the objective is to resolve the issue that objects outside the measurement or middle electrode plane affect the reconstructed images because of the fringe effect. As shown in Figure 7a–c, three distributions were simulated, i.e., single rod, two rods, and three rods. The influence of the length and width of the rods is evaluated for all the selected distributions. The gap between adjacent electrode planes was also varied so that the optimal gap can be determined. Among these three distributions, the single rod or two rods are outside the middle electrode plane. In Figure 7c, the axial distribution of three rods is similar to that in Figure 5. However, the length of rod inside the measured plane is increased to be the same as the length of ERT sensor. The other two rods outside the measured plane has the same length. To illustrate the changes in fringe effect with the changes in conductivity contrast, two modifications were made in simulation for comparison purpose: (1) Conductive objects (σ = 1 S/m) were employed; (2) the conductivity of background medium was changed to be 0.2 S/m. 

**Figure 8:**
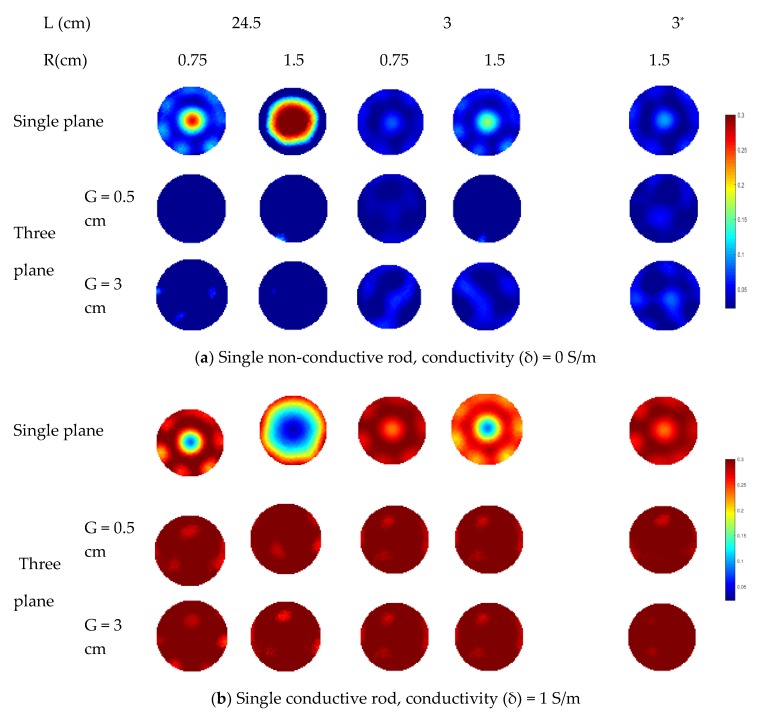
Reconstruction results of single-rod distribution with three-plane ERT sensor or single-plane ERT sensor (* represents the distance between the bottom of a rod and the top end of the middle electrode plane is increased from 0.5 cm to 3 cm) .

In the simulation, the rod length varies from 3 to 24.5 cm while half of the length of the sensor wall is 25 cm. For each specified length, two different rod diameters (i.e., 0.75 cm and 1.5 cm) are simulated for comparison. In Figure 8, the distributions in all cases are reconstructed using the Landweber iteration. With the single-plane ERT sensor, the reconstructed images are consistent with the previous analysis. For the rods outside the sensor plane, they affect the reconstructed images due to the fringe effect. The increase in the rod length and diameter would make the rods more prominent in the reconstructed images, i.e., more severe fringe effect. With the proposed three-plane sensor scheme, the fringe effect is reduced significantly. Good results are obtained when the gap between adjacent electrode planes is 0.5 cm, as discussed in Section 2.

As mentioned previously, the various distances of objects away from the end of the measurement electrode plane suffer from different fringe electric fields. To validate the effectiveness of the three-plane sensor scheme, the distance between the bottom of a rod and the top end of the middle electrode plane is increased from 0.5 cm to 3 cm. In Figure 8, the reconstructed images show that the fringe effect is also reduced with the three-plane sensor scheme in this case.

**Figure 9:**
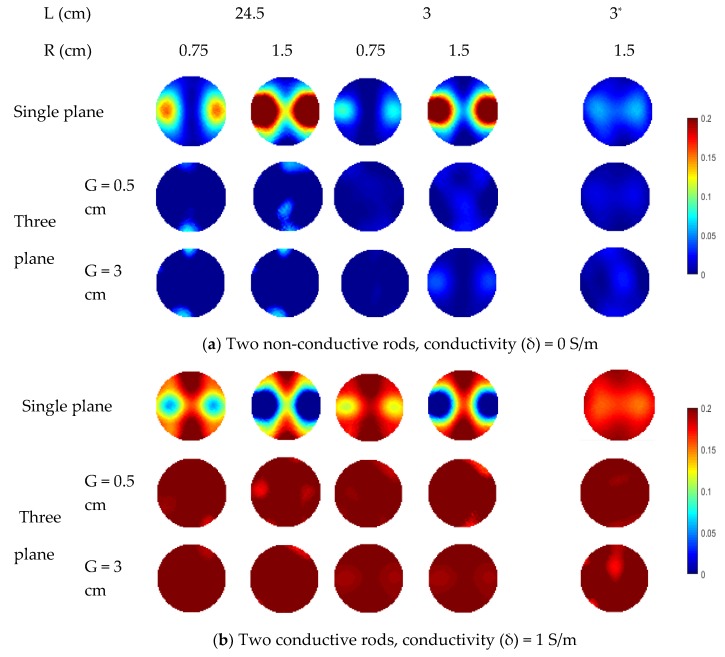
Reconstructed images of two non-conductive or conductive objects with three-plane ERT sensor or single-plane ERT sensor (* represents the distance between the bottom/top of each rod and the top/bottom end of the middle electrode plane is increased from 0.5 cm to 3 cm).

**Figure 10:**
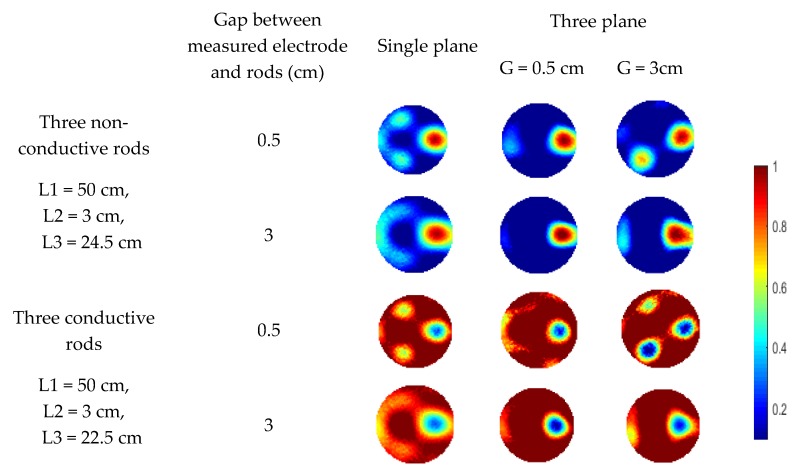
Reconstructed images of three objects with three-plane or single-plane ERT sensor.

For two rods, similar phenomenon is observed in the reconstructed images in Figure 9. The fringe effect induced by the two rods outside the measurement electrode plane is significantly reduced. However, the rods are weakly visible when the length of conductive rods is 3 cm with 3 cm gap between the measurement plane and compensation plane because the compensation of fringe effect is decreased due to the compensation plane was moved away from the measurement plane, which has been validated in the previous section. In Figure 9, although the fringe effect can be reduced when the gap between the electrode plane is increased from 0.5 cm to 3 cm, artefacts can still be observed in some cases. Compared with the previous simulation of three objects, the length of the rods outside the middle plane is changed. Meanwhile, rods of 1.5 cm in diameter are used in this case. Similar set up about the gap between the edge of the measured electrode and the bottom of rods was employed for the rods outside the measured plane. As shown in Figure 10, over-estimation of the rod inside the measurement plane and the fringe effect induced by the outside rods cannot be reduced when the gap between adjacent electrode planes is 3 cm. For conductive rods, artefacts are obvious in the reconstructed images. The fringe effect induced by the rod with longer length cannot be compensated when the gap of the adjacent plane is 3 cm. This phenomenon also proved that the increase in the gap between the measurement plane and compensation plane will lead to the decrease in efficiency of the proposed three-plane sensor scheme.

The reconstructed images represent the local optimum for the corresponding distributions with the single-plane or three-plane ERT sensors. This indicates that the accuracy is improved with the three-plane ERT sensor, compared with the single-plane ERT sensor for the specified object distributions. A smaller gap between the adjacent electrode planes is more suitable for the proposed three-plane sensor scheme. On the other hand, the length, number, and diameter of the objects outside the measurement plane influences the fringe effect, but the three-plane sensor scheme can work properly to reduce the fringe effect in each case. Note that the reconstructed images of non-conductive objects are good, while the reconstructed images of conductive ones suffer from artefacts. Compared with the non-conductive objects, the conductive objects can attractive more electric field lines. Severe distortion of fringe electric fields means that the axial distance between two adjacent electrode planes cannot be too large. Otherwise, the fringe effect sensed by these two electrode planes would be very different, making the compensation be ineffective. This gives a guidance on the design of the multi-plane ERT sensor, i.e., with a sufficiently small distance between adjacent electrode planes.

### 4.2. Two-Plane ERT Sensor Scheme

The proposed three-plane sensor scheme would have some issues in practice. Three electrode planes increase the complexity of the sensor design. More measurement channels are needed, which is related to the data acquisition hardware. Therefore, it is meaningful to investigate the performance of two-plane sensor scheme on reducing the fringe effect. For the two-plane ERT sensor scheme, the electrode plane below the middle plane in the three-plane sensor scheme is removed with only one auxiliary plane for the compensation of fringe effect. According to the above simulation results, the fringe effect induced by outside objects may be compensated with the measurements in the auxiliary electrode plane. Compensation can be made by adaptively using the method proposed for the three-plane sensor scheme, taking the following form:

(10) $$V_{ac}=V_{m}-WF*V_{t}.$$

**Figure 11:**
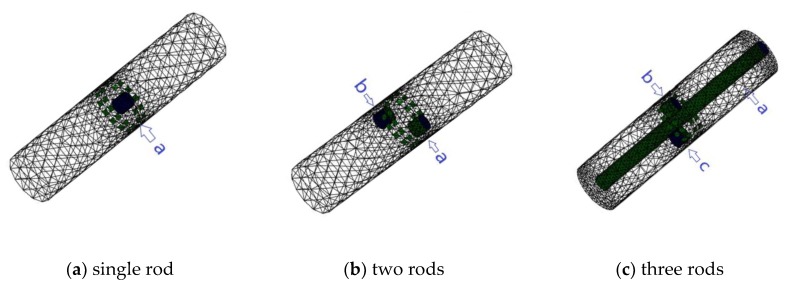
3D view of rods in different axial and cross-sectional positions inside two-plane ERT sensor.

**Figure 12:**
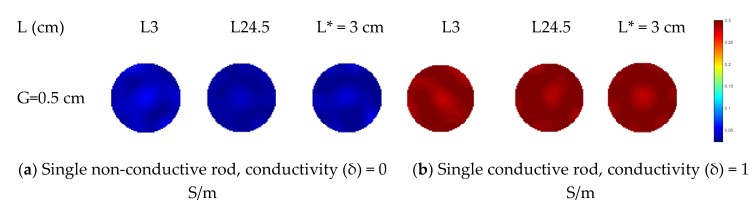
Reconstructed images of single object with two-plane ERT sensor.

As shown in Figure 11, similar cross-sectional object distributions to the previous simulation were used. For every set-up object distribution, rods with the length of 3 cm and 24.5 cm are used for comparison. In Figure 11a, the rod is moved up about by 3 cm. The rod of 3 cm long is used for simulation in this case and it is represented by ‘L*’ in Figure 12. For the object distribution in Figure 11b,c, the rods outside the measurement plane have two different cases, i.e., rods in the same side or opposite side regarding the measured plane. In Figure 11b, one case is placing two same rods above the top end of measurement electrodes by 0.5 cm. Another case is moving the left rods to the opposite side where the rod is below the bottom end of measured electrodes by 0.5 cm. In Figure 11c, some changes were made regarding the lengths of three rods. Firstly, ‘rod a’ is as long as the sensor, which is inside the measurement plane. The other two rods were set up in a similar way to in the two-rods distribution in Figure 11b regarding the axial positions of rods. 

**Figure 13:**
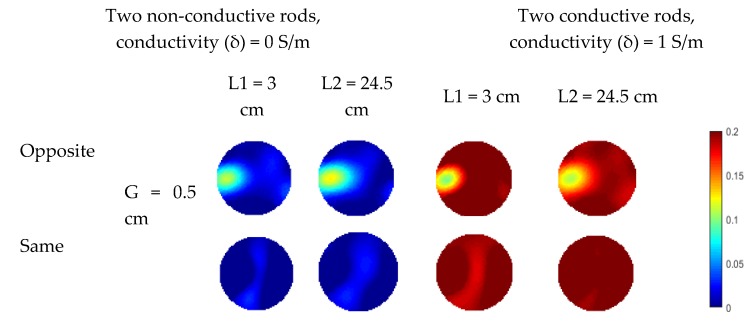
Reconstructed images of two objects with two-plane ERT sensor.

**Figure 14:**
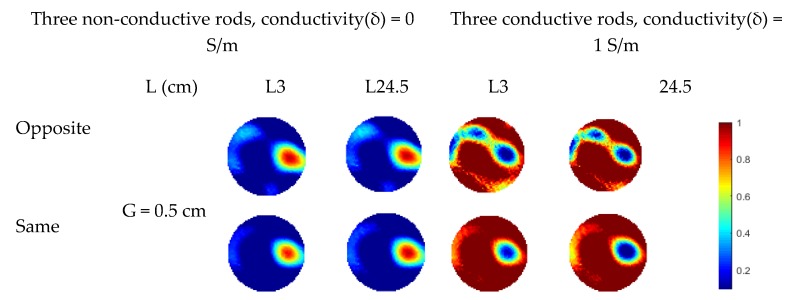
Reconstructed images of three objects with two-plane ERT sensor.

As shown in Figure 12, Figure 13 and Figure 14, the reconstructed images of the distributions in Figure 7a–c are obtained using the two-plane sensor scheme. The quality of the reconstructed images decreases significantly after compensation with the two-plane sensor scheme. Specifically, artefacts can be observed in the reconstructed images of single rod, especially in the area of the placed rod. For the reconstructed images of two rods, the rod below the measurement plane affects the image. This phenomenon can be observed in the reconstructed images of three rods. Therefore, for the proposed two-plane sensor scheme, the compensation plane cannot compensate properly the fringe effect induced by the object, which is placed below the measured plane if the compensation plane is above the measurement plane. The fringe effect induced by the conductive objects causes severe artefacts in the reconstructed images. The effect is more obvious than that of three-plane sensor scheme. The two-plane sensor scheme is not affected by the variation in fringe effect due to changes in the length, diameter, and number of objects as well. The simulation results show that the proposed multi-plane sensor scheme can compensate for the fringe effect of ERT sensor, providing more accurate images of real object distributions inside the measurement plane.

## 5. Experiment and Results

An experimental system with a three-plane ERT sensor and a two-plane plane sensor is established to verify the simulation results. In this ERT system, the three-plane ERT sensor has three identical electrode planes with 3 cm gap between adjacent ones, while the gap between adjacent electrode plane of the two-plane ERT sensor is 2 cm. For the multi-plane ERT sensors, current sources are used to inject currents into multiple pairs of electrodes in the multiple electrode planes at the same time. Each current has a peak-to-peak magnitude of around 1 mA and nearly out of phase. The signal frequency of the injected AC current is 10 kHz for both the three-plane and two-plane sensor scheme. By multiplexing, each current is injected into a pair of adjacent electrodes in the corresponding electrode plane. The potential difference between each possible pair of adjacent electrodes in each electrode plane is conditioned with a differential amplifier (amplified by 100 times through two stages with each stage 10 times) and then measured by a data acquisition unit. Each measurement is sent to a PC for image reconstruction via USB. Finally, an image is reconstructed in MATLAB using the received data. 

### 5.1. Three-Plane ERT Sensor

**Figure 15:**
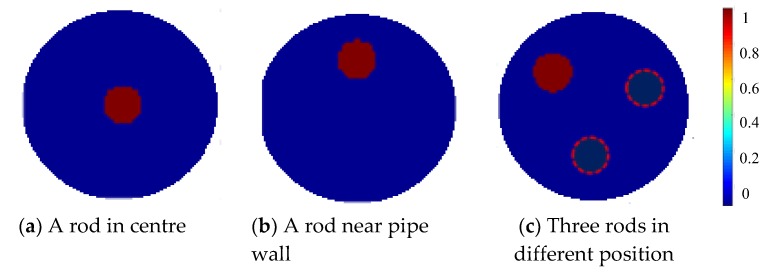
Normalized object distributions tested with three-plane and two plane ERT sensor in experiment.

For the three-plane sensor scheme, three similar object distributions as in the initial simulation were setup in the experiment, i.e., a rod in center, a rod near pipe wall, and three rods in different axial and cross-sectional positions. The cross-sectional views of the normalized true distributions in the three setups are shown in Figure 15. All the rods are non-conductive with saline of conductivity 0.023 S/m as the background medium. These cylindrical rods for imaging have a diameter of 3 cm and a length of 20 cm. Note that in Figure 15c, only the rod inside the middle plane is shown in solid filling, with a length of 20 cm. The other two rods (dotted circles) have the same diameter of 3 cm and a length of 8 cm and placed above and below the middle plane, respectively, and at different cross-sectional positions as in Figure 5. They are away from the top or bottom ends of the electrodes in the middle plane by around 2 mm, respectively.

**Figure 16:**
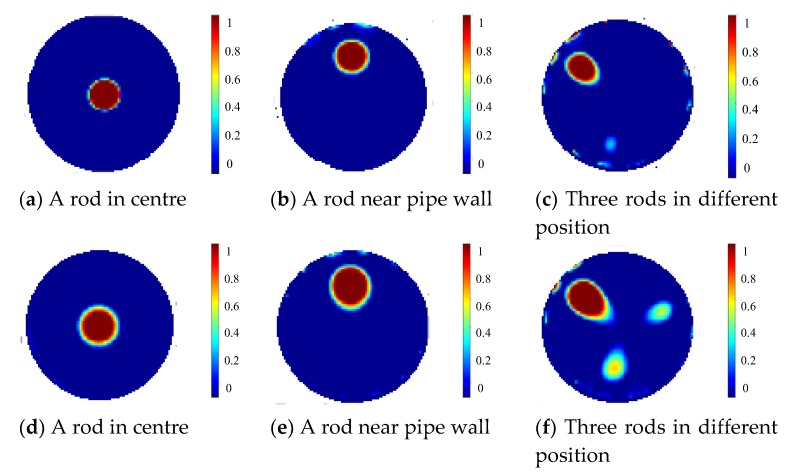
Reconstruction results for three experimental setups with three-plane ERT sensor (**a**–**c**) or single-plane ERT sensor (**d**–**f**).

With the proposed three-plane ERT sensor scheme, the reconstruction results of these three setups using the projected Landweber iterations are shown in Figure 16a–c. For comparison, the reconstruction results with a single-plane ERT sensor are shown in Figure 16d–f. Note that the single-plane ERT sensor only consists of the middle electrode plane in the three-plane ERT sensor. The weighting factors are chosen to be 0.05, 0.09, and 0.03 for the setups in Figure 15a–c, respectively, during the reconstruction. The relaxation factor is updated in each iteration according to the linear search method proposed by Liu et al. [15]. Figure 16 shows that the three-plane ERT sensor scheme can reduce over-estimation by the Landweber iteration for all the distributions and the fringe effect induced by objects outside the sensor plane, improving the quality and accuracy of reconstructed images significantly. This is consistent with the conclusions drawn from the previous simulation.

### 5.2. Two-Plane ERT Sensor

**Figure 17:**
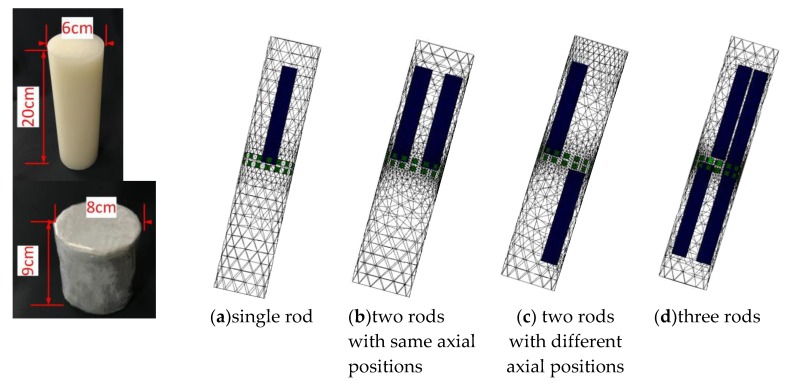
Four distributions setup in experiment with two-plane or single-plane ERT sensor.

For the two-plane sensor scheme, four object distributions were established in the experiment, i.e., single rod in the center, two rods, and three rods in different cross-sectional and axial locations. 3D views of the true object distributions are shown in Figure 17. In the two-plane ERT sensor, the bottom electrode plane is chosen to be the measurement plane, while the top plane is used to compensate for the fringe effect. Cylindrical non-conductive rods were used to setup the above object distributions, and the background medium is tap water. In Figure 17, nylon rods and sand-filled rods have different diameters, which are 6 cm and 8 cm, respectively, while the lengths of these two kinds of rods are 20 cm and 9 cm, respectively. In Figure 17a, the axial position of nylon rod is away from the top end of the electrodes in the bottom plane by around 2 mm. In Figure 17b, two nylon rods of 20 cm long are placed near the pipe wall, and their axial positions are the same as that of the single rod. In these two cases, the rods are outside the measured plane. In Figure 17c, nylon rods are replaced by sand-filled rods because of the length restriction of the used sensor. One sand rod of 9 cm long is placed below the bottom end of the electrodes in the measurement plane by around 2 mm, while another rod of the same length is above the top end of the measurement electrode by around 2 mm. The cross-sectional positions of two rods are the same as in Figure 17b. In Figure 16d, a nylon rod is placed inside the middle plane. Two sand-filled rods are placed outside the measurement plane. The axial positions of those two rods are the same as in Figure 17c.

**Figure 18:**
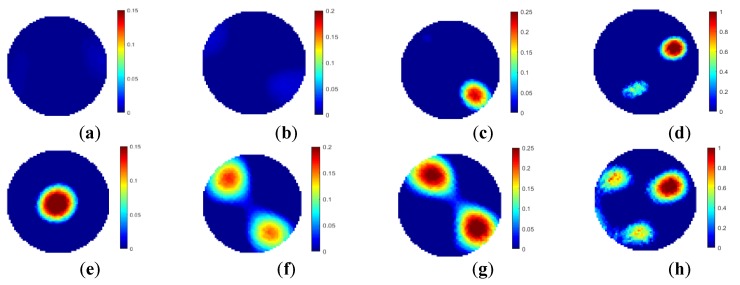
Reconstruction results for four experimental setups with two-plane ERT sensor (**a**–**d**) or single-plane ERT sensor (**e**–**h**).

Figure 18 shows the reconstructed images of the specified object distributions with the proposed two-plane sensor scheme. The reconstructed images with the conventional single-plane ERT sensor are also displayed for comparison. According to Figure 18, the two-plane ERT sensor scheme can reduce the fringe effect induced by objects outside the measurement sensor plane and over-estimation by the Landweber iteration. The weighting factor are determined to be 0.31, 0.26, 0.21, and 0.18 for the imaging scenarios in Figure 18a–d respectively. However, it does not work when the objects are below the measurement plane, if the auxiliary electrode plane used for compensation is above the measurement plane. The experiment results are consistent with the simulation results. On the other hand, the image quality with the two-plane sensor scheme is not as good as that of the three-plane sensor scheme.

**Figure 19:**
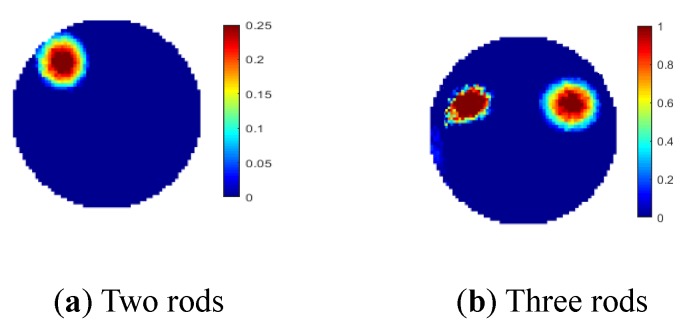
Reconstruction results for two experimental setups (Figure 17c,d) with two-plane ERT sensor by altering the measurement plane and compensation plane.

Unlike the three-plane sensor scheme, the two-plane sensor scheme can adjust the role of measurement plane and the compensation plane. It means that every electrode plane of the two-plane ERT sensor can be the measurement plane or compensation plane, depending on the practical situation. For the experimental setups Figure 17c,d, the images are reconstructed by changing the measurement plane to the compensation plane. Meanwhile, the compensation plane changes to the measurement plane, as shown in Figure 19. The weighting factor are determined to be 0.275 and 0.237. The reconstruction results are consistent with previous findings. For the top rod in both cases, images were reconstructed. The over-estimation of its size is reduced as the its position change from the outside of the measurement plane to the inside of it. On the contrary, the bottom rod does not affect the image because of the fringe effect induced by it was compensated by the compensation plane. The flexibility of two-plane senor scheme has been proved.

## 6. Conclusions

This paper presents multi-plane ERT sensor schemes, three-plane and two-plane, to compensate for the fringe effect induced by objects outside the measurement sensor plane. Both simulation and experimental results validate the proposed method by considering the influence of the length, diameter, and axial position of objects on the fringe effect with respect to several object distributions. During image reconstruction, the objects outside the measurement plane are almost invisible in the reconstructed images using the multi-plane ERT sensor schemes. Meanwhile, it is found that the Landweber iteration would over-estimate the size of nonconductive object in the case of axially uniform distributions, due to the large conductivity contrast between the object and the background, which can be alleviated by the multi-plane sensor schemes. However, this may be not the case when the contrast becomes smaller, e.g., imaging two-phase flows with both phases conductive. In this case, other reconstruction algorithms may be applied to compensate for the reduction in the size of objects to be imaged, caused by the compensation for the fringe effect. Finally, the gap between the adjacent electrode planes should be sufficiently small so that the multi-plane sensor scheme can reduce the fringe effect effectively. It is important to determine the weighting factors for compensation, because all the weighting factors used previously are determined empirically. In the future, it is necessary to select the weighing factor adaptively for practical applications, which may be accomplished by a linear search method. Further simulation and experiments are needed to investigate the effectiveness of the proposed method in more complicated scenarios. For multiphase flow measurement, the proposed compensation method can be applied to reduce the fringe effect induced by the axially non-homogeneous distribution of dispersed phase, e.g., slug flow and plug flow are common in industrial processes. Due to the fringe effect, erroneous images of bubble columns will be obtained if large bubbles are outside the measurement electrode plane. With the proposed method, the quality of 2D ERT imaging can be improved to meet the measurement requirements in practical industrial applications.

## Figures and Tables

**Table 1:** Relative image errors and correlation coefficients of Figure 6a–f regarding respective true distribution.

Subfigure Number	(a)	(b)	(c)	(d)	(e)	(f)
**Relaxation number**	α is updated in each iteration by linear search					
**Number of Iterations**	216	18	16	2	2	2
**Relative image errors (%)**	6.7	34	57	57	65	93
**Correlation coefficient (%)**	99.7	93	81.8	85	81	7.3
